# Dynamic Changes in Phase-Amplitude Coupling Facilitate Spatial Attention Control in Fronto-Parietal Cortex

**DOI:** 10.1371/journal.pbio.1001936

**Published:** 2014-08-26

**Authors:** Sara M. Szczepanski, Nathan E. Crone, Rachel A. Kuperman, Kurtis I. Auguste, Josef Parvizi, Robert T. Knight

**Affiliations:** 1 Department of Psychology, University of California, Berkeley, Berkeley, California, United States of America; 2 Helen Wills Neuroscience Institute, University of California, Berkeley, Berkeley, California, United States of America; 3 Department of Neurology, Epilepsy Center, Johns Hopkins Medical Institutions, Baltimore, Maryland, United States of America; 4 Department of Neurology, Children's Hospital and Research Center, Oakland, Oakland, California, United States of America; 5 Department of Surgery, Division of Neurological Surgery, Children's Hospital and Research Center, Oakland, Oakland, California, United States of America; 6 Department of Neurological Surgery, University of California, San Francisco, San Francisco, California, United States of America; 7 Laboratory of Behavioral and Cognitive Neurology, Department of Neurology and Neurological Sciences, Stanford University, Stanford, California, United States of America; 8 Stanford Human Intracranial Cognitive Electrophysiology Program (SHICEP), Stanford University, Stanford, California, United States of America; University of Oregon, United States of America

## Abstract

Attention is a core cognitive mechanism that allows the brain to allocate limited resources depending on current task demands. A number of frontal and posterior parietal cortical areas, referred to collectively as the fronto-parietal attentional control network, are engaged during attentional allocation in both humans and non-human primates. Numerous studies have examined this network in the human brain using various neuroimaging and scalp electrophysiological techniques. However, little is known about how these frontal and parietal areas interact dynamically to produce behavior on a fine temporal (sub-second) and spatial (sub-centimeter) scale. We addressed how human fronto-parietal regions control visuospatial attention on a fine spatiotemporal scale by recording electrocorticography (ECoG) signals measured directly from subdural electrode arrays that were implanted in patients undergoing intracranial monitoring for localization of epileptic foci. Subjects (*n* = 8) performed a spatial-cuing task, in which they allocated visuospatial attention to either the right or left visual field and detected the appearance of a target. We found increases in high gamma (HG) power (70–250 Hz) time-locked to trial onset that remained elevated throughout the attentional allocation period over frontal, parietal, and visual areas. These HG power increases were modulated by the phase of the ongoing delta/theta (2–5 Hz) oscillation during attentional allocation. Critically, we found that the strength of this delta/theta phase-HG amplitude coupling predicted reaction times to detected targets on a trial-by-trial basis. These results highlight the role of delta/theta phase-HG amplitude coupling as a mechanism for sub-second facilitation and coordination within human fronto-parietal cortex that is guided by momentary attentional demands.

## Abstract 2

Electrocorticography reveals how coupling between two frequencies of neuronal oscillation allows the frontal and parietal areas of the cortex to control visual attention from moment to moment in the human brain.

## Author Summary

The frontal and parietal areas of the cortex control the ability to focus visuospatial attention, and damage to these areas results in profound attentional disturbances. Although much research has concentrated on *where* these areas are located, little is known about *how* these areas may function in humans. Previous studies have demonstrated that neuronal spiking is more likely to occur in specific time windows based upon the phase of lower frequency neural oscillations – rhythmic or repetitive neuronal activity. These low-frequency rhythms are hypothesized to coordinate the timing of neuronal firing within local and across network regions. Here, we investigated how human frontal and parietal cortices use neural oscillations to control visuospatial attention. We identified a high-frequency component of electrical brain activity, broadband high gamma (70–250 Hz) amplitude, that became phase-locked to a slower rhythm, delta/theta (2–5 Hz), over frontal, parietal, and visual areas while the study subjects paid attention to the peripheral visual field. Changes in the strength of the coupling between delta/theta phase and high gamma amplitude predicted the attentional behavior of the subjects across single trials. From these results, we conclude that coupling between delta/theta phase and high gamma amplitude serves to coordinate information within – and perhaps between – frontal and parietal areas during allocation of visuospatial attention.

## Introduction

Attention, a critical component of perception and goal-directed behavior, allows the brain to allocate its limited resources depending on current task demands. A number of areas located in frontal and posterior parietal cortex (PPC), often referred to collectively as the fronto-parietal attention network, are crucial for controlling the attentional selection process in both humans and non-human primates [1]–[5]. These areas include the intraparietal sulcus (IPS) and superior parietal lobule (SPL) in PPC as well as portions of superior-lateral precentral cortex, also known as the frontal eye fields (FEF), and dorsal medial frontal cortex, also known as the supplementary eye field (SEF) [1],[4]. In the human brain, the temporal-parietal junction (TPJ) and portions of the inferior, middle, and superior frontal gyri are also involved during attentional allocation [1].

Attentional control is often investigated in these regions using functional magnetic resonance imaging (fMRI) methods (i.e., investigating BOLD time courses in each region) or electrophysiological methods (single-unit, multi-unit, or local field potential [LFP] recordings in macaques or with electroencephalography [EEG] in humans). fMRI has excellent spatial resolution and can identify functionally active networks, but is constrained by the low temporal resolution of the blood-oxygen-level dependent (BOLD) signal. Electrophysiological recordings in monkeys provide excellent temporal resolution, but cannot easily be used to simultaneously investigate an entire network of areas. Scalp EEG studies (see [6],[7] for reviews) have provided important insights into the understanding of attention, but EEG has spatial limitations due to the inverse problem. A handful of studies have attempted to solve the inverse problem using EEG or magnetoencephalography (MEG) combined with source localization techniques to investigate the spatial attention network in greater detail [8],[9], but relatively little knowledge exists about how fronto-parietal areas support attentional behavior on a fine spatiotemporal scale in the human brain.

Populations of neurons oscillate together and synchronize their firing and post-synaptic potentials in a rhythmic fashion [10]. These oscillations emerge in multiple frequency bands: delta (1–4 Hz), theta (4–8 Hz), alpha (8–12 Hz), beta (12–30 Hz), and low gamma (30–60 Hz). It is also possible to capture neuronal activity by sampling power fluctuations in a broadband high-frequency range, known as high gamma (HG; 60–250 Hz), as studies have shown that HG activity correlates with local spiking activity [11],[12]. Interactions within or between these bands and/or broadband HG have been proposed to serve as mechanisms for coordination within and between brain networks engaged in cognitive processing [13]–[15].

Phase-amplitude cross-frequency coupling (PAC) is a mechanism that has been proposed to coordinate the timing of neuronal firing within local neural networks (see [16] for a review). PAC, the statistical dependence between the phase of a low-frequency rhythm and the amplitude (or power) of the high-frequency component of electrical brain activity, is proposed to operate through a physiologically plausible mechanism: low-frequency phase controls neuronal excitability through fluctuations in membrane potentials, while increases in high-frequency power reflect increases in local neuronal spiking activity [11],[12]. Previous studies have demonstrated that the probability of neuronal spiking changes with the phase of the lower frequency rhythm and can define time windows during which neurons are more or less likely to fire [16]–[18]. Thus, it has been hypothesized that these lower-frequency oscillations (i.e., in the delta, theta, alpha, or beta bands) coordinate information among areas by modulating the excitability within local neuronal ensembles [14],[17],[18]. Canolty and colleagues [19] first demonstrated that the phase of the ongoing theta oscillation is coupled to increases in HG (>70 Hz) power across a range of motor and language tasks in humans. PAC has since been reported between numerous different frequency bands within human cortex and subcortical structures [20]–[23]. To our knowledge, no study to date has investigated if PAC may serve as a mechanism for visuospatial attention control within human fronto-parietal cortex.

We measured the neural dynamics and frequency-band interactions within regions of the human fronto-parietal attention network during allocation of spatial attention using a dynamic spatial-cuing task [24]. We collected electrocorticography (ECoG) recordings, measured directly from subdural electrode arrays implanted in patients undergoing intracranial monitoring for localization of epileptic foci, in order to examine the role of PAC as a mechanism for coordination within the fronto-parietal network, which adjusts parameters on a millisecond basis depending on momentary attentional demands.

## Results

**Figure 1:**
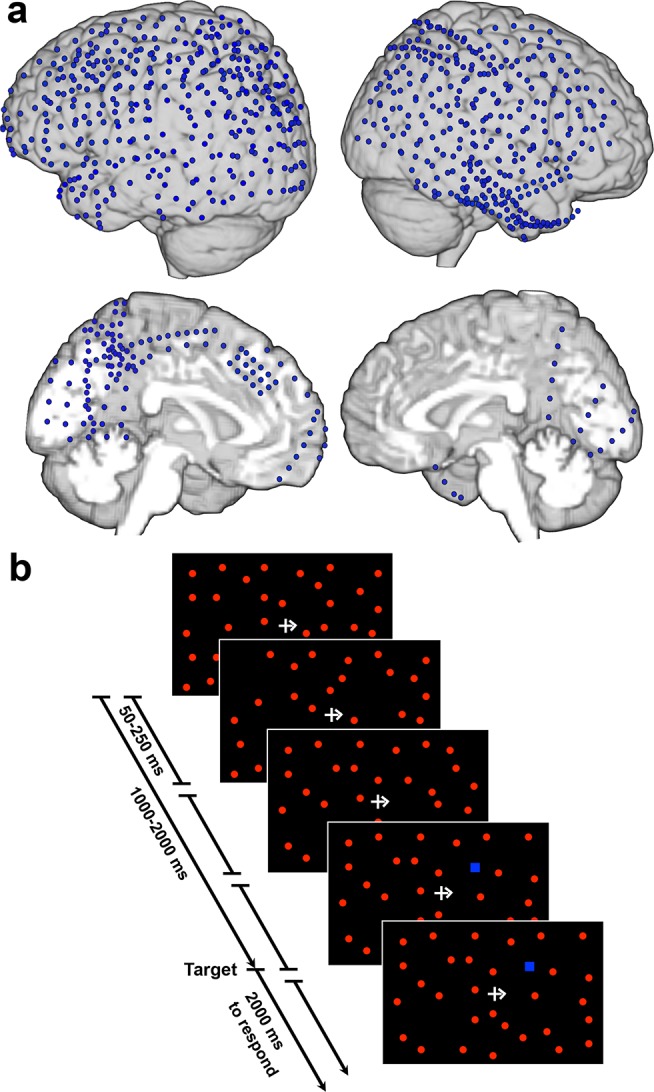
Electrode coverage and Starry Night Test (SNT). (**a**) Overlap of implanted electrodes across all subjects (*n* = 8) on the lateral and medial surfaces (top, bottom, respectively) in the right and left hemispheres (right, left, respectively) overlaid on a cortical reconstruction of the MNI standardized brain. (**b**) In the SNT, subjects allocated their visual attention to either the RVF or LVF, as indicated by a cue at fixation, and waited for a target (blue square) to appear somewhere in the visual field. Targets appeared on a dynamic background of red circle distracters. Subjects responded with a button press once they detected the target. Example of a single trial is shown during which the RVF was cued.

We collected ECoG recordings from eight subjects with extensive lateral and medial frontal, parietal, and occipital cortex coverage encompassing either the left or the right hemisphere (LH, *n* = 5, RH, *n* = 3; Figure 1a; Table 1) while they performed a modified version of a dynamic reaction time (RT) task (Starry Night Test [SNT] [24]). In this task, subjects were instructed to maintain fixation on a central cross and were cued to allocate their attention to either the right or left visual fields (RVF, LVF), to wait for the appearance of a blue target that could appear any time between 1000–2000 ms following trial onset and was embedded among numerous flickering visual distractors (giving the SNT its name), and to respond once the target was detected (Figure 1b). Accuracy was high (d-prime: 5.03±0.68 [average ± standard error of the mean (SEM)]; hits: 97.21%±1.13; correct rejections: 98.18%±0.38), and subjects responded to targets in an average of 601.31±28.64 ms. Neither accuracy (*p*>0.40) nor RTs (*p*>0.20) differed between trials where the target appeared contralateral or ipsilateral to the implanted hemisphere. However, a significant linear trend was revealed across eccentricity locations when RTs were collapsed across visual fields, wherein subjects responded most quickly to targets that appeared in parafoveal locations (576.83±36.46 ms), slower to targets in the middle of the visual field (598.83±38.33 ms), and the slowest to peripheral targets (621.49±40.97 ms), *F*(1,7) = 13.22, *p*<0.01. This finding is consistent with previous studies demonstrating that target distance from the fovea impacts the speed with which subjects can allocate their locus of attention [25].

**Table 1:** ECoG subjects.

Subject	Sex	Age	Handedness	Hemisphere Coverage	Area Coverage	Hospital
S1	M	20	L	LH	LF, P, T	Johns Hopkins Hospital
S2	M	18	R	LH	Md, O, P, T	Children's Hospital, Oakland
S3	M	41	R	LH	LF, Md, P	Stanford Hospital
S4	M	45	R	RH	Md, O, P, T	Stanford Hospital
S5	M	24	R	RH	LF, O, P, T	Stanford Hospital
S6	F	24	R	RH	LF, P, T	Stanford Hospital
S7	F	21	R	LH	LF, Md, O, P	Stanford Hospital
S8	F	52	R	LH	Md, O, P, T	Stanford Hospital

F, female; M, male; L, left; R, right; LH, left hemisphere; RH, right hemisphere; LF, lateral frontal; Md, medial; O, occipital; P, parietal; T, temporal.

### HG Power Tracks Visuospatial Attention

**Figure 2:**
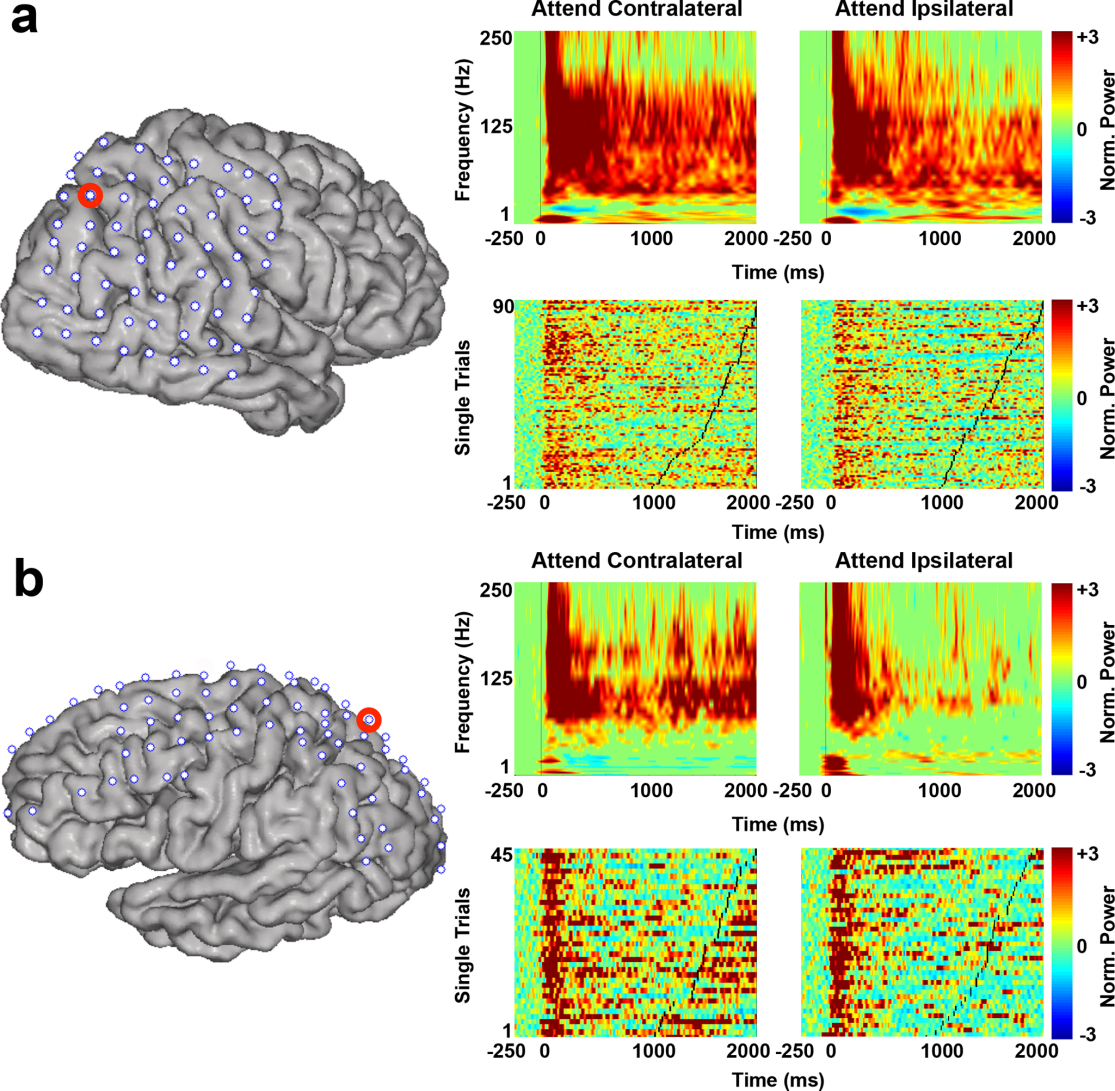
HG power changes track attentional allocation. Event-related spectral perturbations (ERSPs; top) and vertically-stacked single-trial HG traces (bottom) in two example electrodes of two different subjects, S4 (**a**) and S7 (**b**). The red circle on each subject's cortical reconstruction indicates the electrode from which each of the ERSPs and single-trial HG traces was taken. ERSPs were averaged across trials when subjects attended to either the visual field contralateral (left) or ipsilateral (right) to the implanted hemisphere. The normalized power from 1–250 Hz is shown time-locked to the beginning of each trial. HG power (70–250 Hz) is shown for each individual trial for contralateral (left) and ipsilateral (right) attentional conditions. Traces are locked to each trial onset and black tick marks signify target onset. Trials are stacked according to target onset.

To determine which electrodes were responsive to spatial attention, we first investigated changes in power across a wide range of frequencies in response to attentional allocation to either the contralateral or the ipsilateral visual field (i.e., the visual field contralateral or ipsilateral to the implanted hemisphere). In a number of electrodes (263 out of 875 electrodes; 30.06%) across eight subjects, we found significant increases in broadband HG power (70–250 Hz; all *p*<0.05, corrected) that were time-locked to trial onset (following each cue). HG power remained elevated throughout the period when subjects were allocating their spatial attention in wait for the target appearance (0–1000 ms post-trial onset). These HG power increases were observed in electrodes covering PPC, lateral, and medial frontal cortex, occipital cortex, and posterior ventral temporal cortex (see Figure 2 for event-related spectral perturbations [ERSPs] and vertically stacked single-trial HG traces in two example electrodes across two separate subjects [S4, S7]). A subset of these electrodes (47 of 263; 17.87%; in seven subjects) exhibited greater HG power increases when subjects allocated attention to the contralateral than to the ipsilateral visual field (all *p*<0.05, corrected). In comparison, only three out of 263 electrodes (1.14%; in one subject) exhibited greater HG power increases when subjects allocated attention to the ipsilateral than to the contralateral visual field (all *p*<0.05, corrected).

Increases in HG power were sometimes accompanied by power increases in lower frequencies (delta/theta: 2–5 Hz, all *p*<0.05, corrected; Figure 2a) and sometimes not (Figure 2b). 59.32% (156 of 263; in eight subjects) of the electrodes with significant HG power increases also had significant power increases in this lower frequency range. Of the 156 electrodes with significant delta/theta power increases, 17 (10.90%) overlapped with the set of 47 electrodes that showed visual field-dependent attentional increases in HG power.

A number of electrodes (119 out of 875 electrodes; 13.60%; in eight subjects) also exhibited significant decreases in alpha and beta power (10–30 Hz; all *p*<0.05, corrected) compared to baseline. This alpha and beta suppression was sustained for 500 ms (e.g., Figure 2a) to 1000 ms or longer (e.g., Figure 2b) following trial onset. All electrodes with sustained alpha/beta suppression were located either in parietal or visual cortex. A subset of these electrodes (21 of 119; 17.65%; in seven subjects) exhibited larger decreases in alpha/beta power when subjects allocated attention to the contralateral than to the ipsilateral visual field (all *p*<0.05, corrected). In comparison, 2 out of 119 electrodes (1.68%; in one subject) exhibited larger decreases in alpha/beta power when subjects allocated attention to the ipsilateral than to the contralateral visual field (all *p*<0.05, corrected). These results are consistent with the notion that decreases in alpha power, which are strongest in the hemisphere contralateral to a stimulus, represent a release from functional inhibition within the visual system [26].

Taken together, the results suggest that power increases in broadband HG and the delta/theta bands, as well as power decreases in the alpha/beta bands, track the allocation of visuospatial attention in human frontal, posterior parietal, and visual cortex.

### PAC Facilitates Visuospatial Attention in Localized Fronto-Parietal Regions

**Figure 3:**
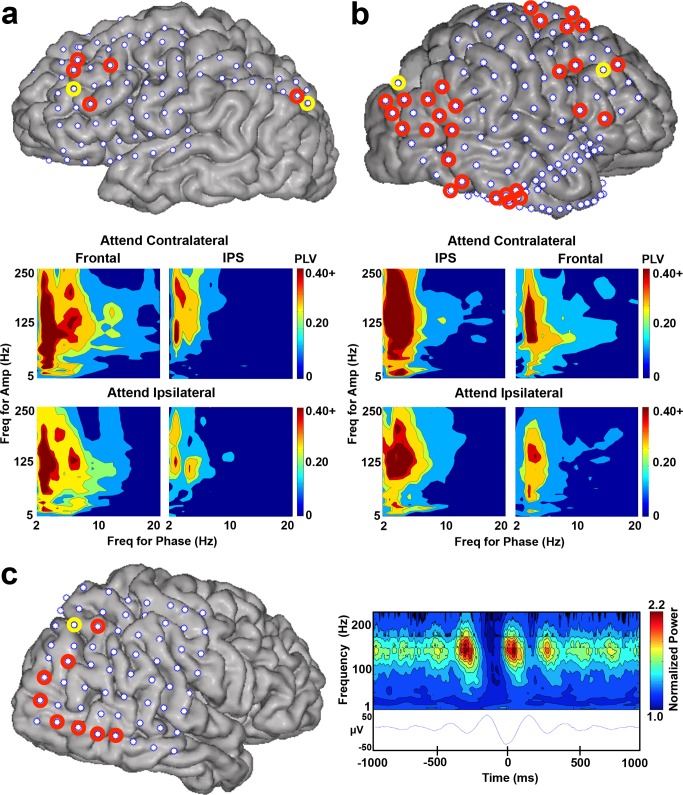
Delta/theta phase-HG amplitude coupling during attentional allocation. Examples of electrodes with significant delta/theta phase-HG amplitude coupling during attentional allocation. (**a,b**) Comodulograms for two example electrodes (circled in yellow on each subject's cortical reconstruction), one over lateral frontal cortex and one surrounding the IPS, are shown underneath the respective brain areas of two subjects, S3 (**a**) and S5 (**b**). Each comodulogram illustrates PAC strength (measured with PLV) across a range of frequencies (x-axis = frequency for phase signal, y-axis = frequency for amplitude signal). Contour lines represent *p*-values (outer to inner: *p* = 0.50, 0.10, 0.05, 0.01, 0.005). Separate comodulograms were calculated for the contralateral and ipsilateral attention conditions. Additional electrodes with significant delta/theta-HG PAC in these two subjects are circled in red. See Figure 6 for the significant electrodes in the remaining subjects. (**c**) A trough-locked spectrogram from an example electrode over PPC (circled in yellow on the cortical reconstruction of S4). Right, bottom: the trough-locked ERP of the filtered delta/theta signal (2–5 Hz). Right, top: normalized power across a range of frequencies for the corresponding time points of the trough-locked delta/theta signal (below). Additional electrodes with significant delta/theta-HG PAC in S4 are circled in red.

Because previous studies have demonstrated that frequency bands can interact in behaviorally meaningful ways [19],[27], we assessed whether any relationships existed among the different frequency bands (delta, theta, HG) during attentional allocation. We examined PAC between a wide range of frequencies for phase data (2–20 Hz) and for amplitude data (5–250 Hz) (Figure 3a,b) in all of the electrodes that had significant increases in HG power (70–250 Hz) following attentional allocation compared to baseline. In many of these electrodes (123 of 273; 45.05%; in eight subjects), we found significant coupling between the phase of the delta/theta signal (2–5 Hz) and HG amplitude (80–250 Hz) during attention to the cued visual field while awaiting target appearance (0–1000 ms post-trial onset). The coupling values averaged across all attention trials, as measured by the phase-locking value (PLV), ranged between 0.16 and 0.57 for all electrodes showing this significant PAC. We observed delta/theta phase-HG amplitude coupling in electrodes over PPC, especially those surrounding the IPS, lateral and medial frontal cortex, occipital cortex, and posterior ventral temporal cortex (see Figure 3 for examples from three individual subjects (S3–S5) and Figure S1 for the group results plotted on the Montreal Neurological Institute [MNI] brain). Furthermore, this PAC remained significant after controlling for eye movements (S8 was eye-tracked during recording and all trials during which a saccade of 0.50° or larger was made were excluded from further analyses; see Materials and Methods for details). Although a higher percentage of RH electrodes (51.56%; 66 out of 128) exhibited significant delta/theta-HG PAC during attentional allocation than LH electrodes (39.31%; 57 out of 145), this difference was not significant between hemispheres (*U* = 3.00, *p*>0.20). However, interhemispheric differences in electrode coverage and density preclude a strong conclusion regarding left vs. right differences.

Many of the electrodes with significant delta/theta-HG PAC (55 of 123; 44.71%; in seven subjects) had significantly higher coupling when subjects were attending to the contralateral, as compared to the ipsilateral, visual field (all *p*<0.01, corrected). Figure 3a,b shows examples of four electrodes (two surrounding IPS and two over lateral frontal cortex) that have higher PAC in contralateral than in ipsilateral trials. Eighteen of the 55 electrodes with higher PAC in contralateral than in ipsilateral trials also had higher HG power in contralateral than in ipsilateral trials. Although the magnitude of the PAC decreased when subjects attended ipsilaterally, it did not disappear completely, which is likely due to the bilateral receptive field properties of the neuronal populations over which we recorded [28]. None of the electrodes exhibited higher PAC when subjects allocated attention to the ipsilateral than to the contralateral visual field (all *p*>0.10, corrected). Because power increases, and thus the signal-to-noise ratio, can affect PLVs [29], we compared the magnitudes of delta/theta power between attend contralateral and attend ipsilateral trials during the same time period analyzed above. Out of the 55 electrodes in which higher PAC was present when subjects attended contralaterally than when they attended ipsilaterally, only two electrodes in one subject had significantly higher delta/theta power in attend contralateral than attend ipsilateral trials (*p*<0.05, corrected). Thus, power differences between conditions cannot account for the PAC differences that we observed between these same conditions.

To address the possibility that the observed PAC was being driven by an event-related potential (ERP) at trial onset, we repeated the analyses using only the longer trials (i.e., target appearance 1500–2000 ms after trial onset). Only the last 1000 ms prior to the target onset were analyzed, so the first 500 ms, when the ERP was generated, was excluded from every trial. This analysis produced similar PAC results: 107 of 273 electrodes (39.19%; in eight subjects) had significant coupling between delta/theta phase and HG amplitude (Figure S2). All 107 of these electrodes were in the original set of 123 electrodes showing significant delta/theta-HG PAC. This demonstrates that the observed PAC was sustained throughout the trial, rather than driven by a single cycle created by a transient ERP. Additionally, a number of these electrodes maintained significantly higher delta/theta-HG PAC (42 of 123; 34.15%; in seven subjects) when subjects were attending to the contralateral, as compared to the ipsilateral, visual field (all *p*<0.01, corrected) and none of these electrodes showed significant delta/theta power differences between attend contralateral and attend ipsilateral trials (all *p*>0.10, corrected), further demonstrating that the observed laterality effects were not solely driven by transient ERPs.

We next examined to which part of the delta/theta phase signal the HG amplitude was locking by calculating the trough-locked spectrogram for each electrode with significant delta/theta phase-HG amplitude coupling. In a large percentage of electrodes (117 of 123; 95.12%; in eight subjects), the HG power was locked to the trough of the delta/theta signal (see Figure 3c for an example from one electrode in S4). Our results are in agreement with previous studies that found HG locks to the trough of the theta signal [19],[27]. Furthermore, this trough-locking of the HG power was significant for at least three phase cycles of the delta/theta signal (∼600–1500 ms; see Figure 3c for example of three cycles), providing additional evidence that the observed PAC was not solely driven by ERPs.

### Single-Trial PAC Predicts Attentional Allocation

**Figure 4:**
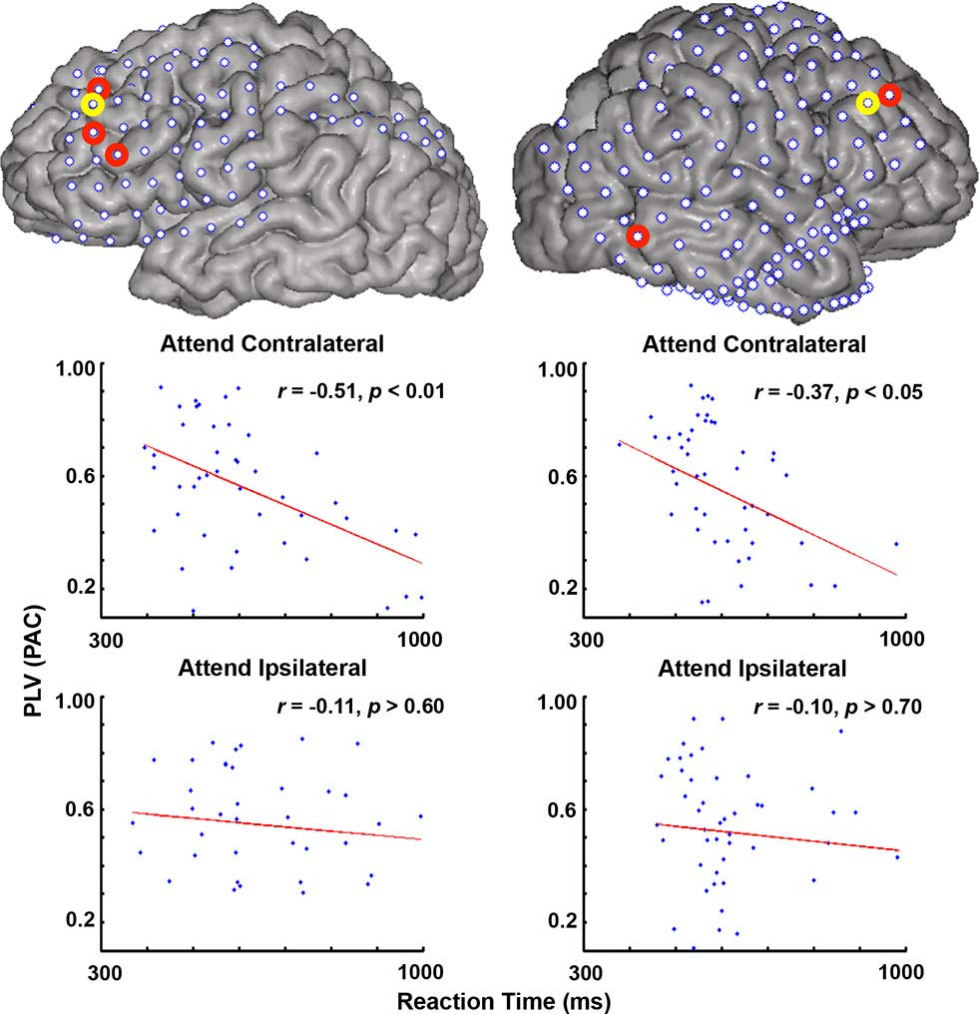
PAC-behavioral correlations. Correlations between the strength of delta/theta-HG PAC (measured with PLV) and reaction times (RTs) during SNT performance. Examples of two different electrodes (circled in yellow on each cortical reconstruction) are shown for two different subjects (S3, left panel and S5, right panel) during attention to either the contralateral (top) or ipsilateral (bottom) visual fields. The red circles on each cortical reconstruction indicate all other electrodes in these two subjects that showed significant PAC-RT correlations across trials. The red line on each scatter plot indicates the regression line through each data set.

Critically, changes in delta/theta phase-HG amplitude coupling were correlated with attentional behavior. Twenty-eight of the 123 electrodes with significant delta/theta-HG PAC also demonstrated significant correlations of PAC with behavior across trials, in which faster RTs were associated with stronger delta/theta-HG coupling, and slower RTs with weaker delta/theta-HG coupling, during the attentional delay period (examples from two subjects, S3 and S5, are shown in Figure 4). The electrodes with significant PAC-behavioral correlations were dispersed throughout frontal (42.86%), parietal (32.14%), and visual cortex (25.00%) across seven subjects. Only the PAC values from trials when subjects attended to the visual field contralateral to electrode implantation correlated with behavior (all *p*<0.05, corrected; Figure 4, S3: *r*(41) = −0.51, *p*<0.01, corrected; S5: *r*(47) = −0.37, *p*<0.05, corrected), whereas the PAC values from trials when subjects attended to the ipsilateral visual field did not reach significance in any of the electrodes (all *p*>0.10, corrected; Figure 4, S3: *r*(40) = −0.11, *p*>0.6, corrected; S5: *r*(47) = −0.10, *p*>0.7, corrected). RTs also did not significantly correlate with mean amplitude of the delta/theta signal (all *p*>0.05, corrected) or with mean amplitude of the HG signal (all *p*>0.10, corrected) across the same time period for either attentional condition in any of these electrodes, suggesting that amplitude changes alone are not driving the results. These findings provide evidence that momentary changes in the strength of delta/theta-HG PAC predict attentional behavior in humans.

### PAC and ERPs Measure Distinct Mechanisms

**Figure 5:**
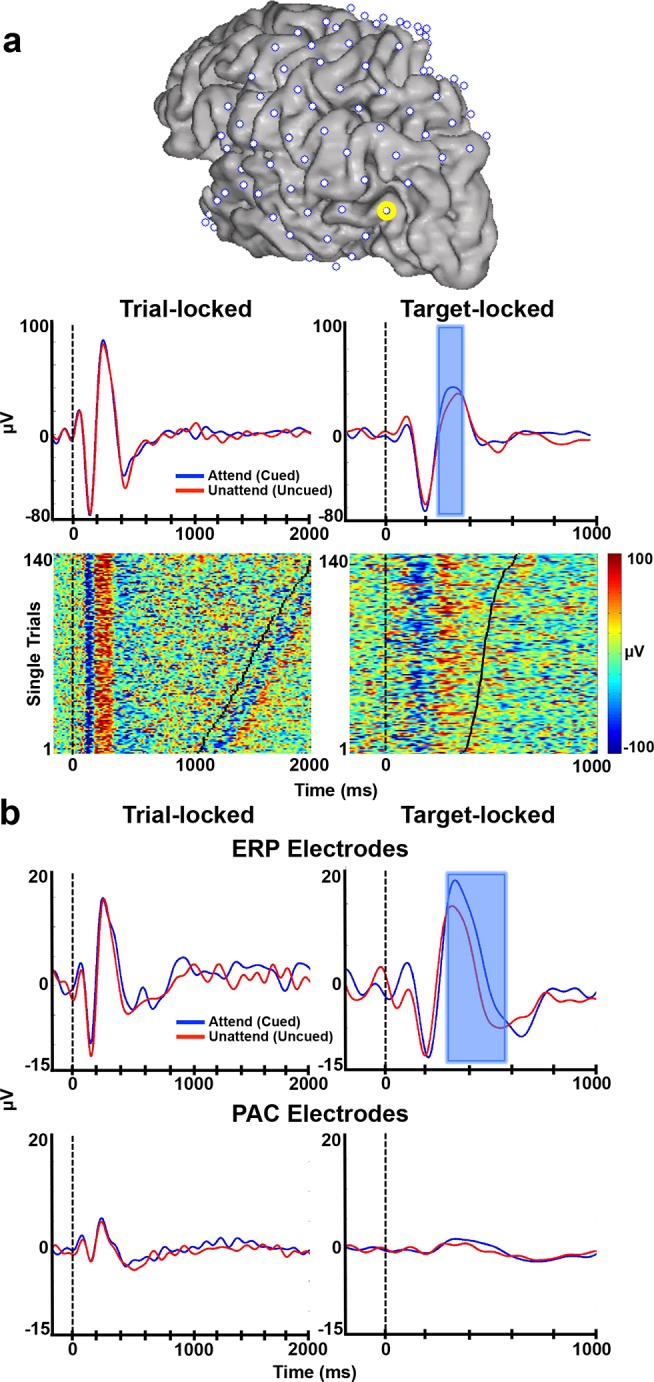
ERPs during attentional allocation. (**a**) Single-trial and averaged ERPs time-locked to trial onset (left) or target onset (right) for an example electrode in a single subject (yellow circle on the cortical reconstruction of S8). Top: ERPs averaged across all trials in which the target appeared in the attended/cued hemifield (blue traces) or in the unattended/uncued hemifield (red traces). Blue shaded region represents the time points during which there was a significant difference between conditions (all *p*<0.05, corrected). Bottom: Single trial ERPs for attention/cued conditions only. Black tick marks signify target onset (left) and manual response (right). Trials are stacked according to target or response onset. (**b**) ERPs averaged across all electrodes that showed an individual ERP (*n* = 77; top) or averaged across all electrodes with significant delta/theta-HG PAC (*n* = 123; bottom) time-locked to trial onset (left) or target onset (right). All other conventions are the same as in (a).

We next examined the relationship between visually evoked ERPs and delta/theta-HG PAC in electrodes across frontal, parietal, and visual cortex, as previous studies have suggested a complex relationship between ERPs and the broadband HG response over visual cortex [30]. We found ERP responses time-locked to the beginning of each trial in 77 of 875 electrodes (8.80%; in eight subjects) and time-locked to the target onset of each trial in 29 electrodes (3.31%; in seven subjects). Each of these ERP responses consisted of the classic visual P1-N1 complex and subsequent P3b [31]–[34]. The P1, N1, and P3b components peaked around 100 ms, 200 ms, and 300 ms post-trial onset, respectively, which is consistent with previous reports of visual responses from ECoG recordings [35],[36]. Figure 5 provides an example of the visual ERPs averaged across trials (attended/cued vs. unattended/uncued; Figure 5a, top) and single-trial ERPs (attended trials only; Figure 5a, bottom) time-locked to trial onset (Figure 5a, left) and target onset (Figure 5a, right) from one electrode in a single subject (S8).

**Figure 6:**
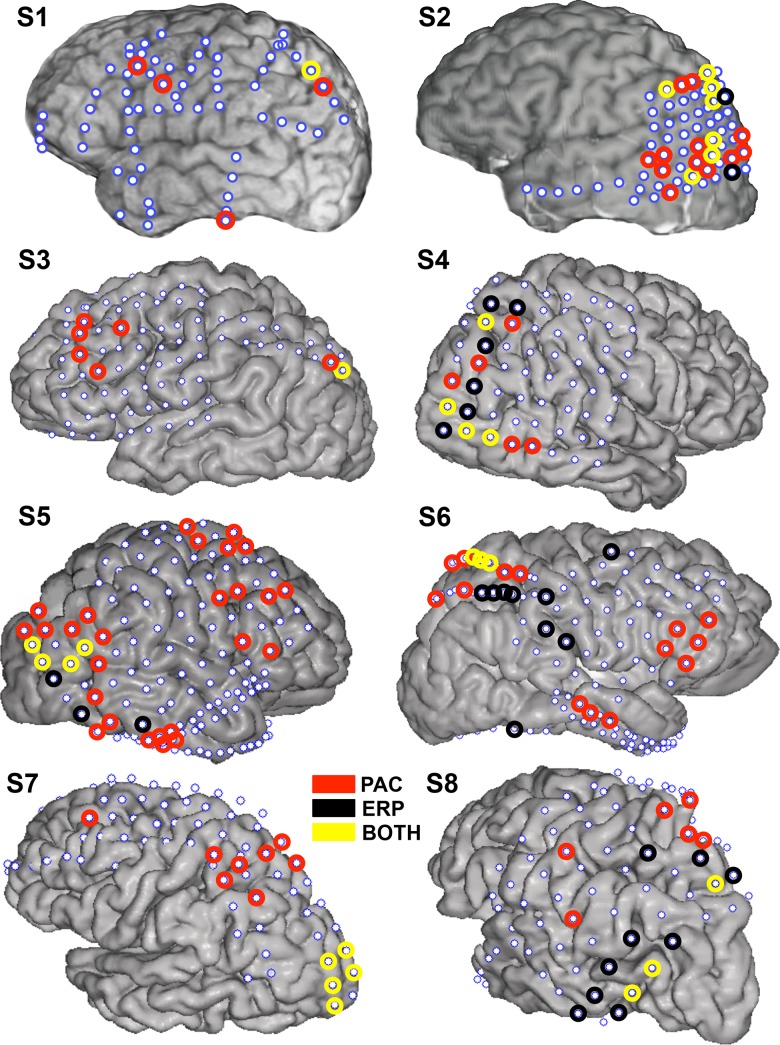
Overlap between delta/theta phase-HG amplitude coupling and ERPs. The electrodes with significant delta/theta phase-HG amplitude coupling (circled in red), trial-locked ERPs (circled in black), or both (circled in yellow) are presented on the cortical reconstructions of each subject (S1–S8). Note that S1, S3, and S6 have limited or no extrastriate coverage.

The majority of electrodes with visually evoked ERP responses were located over visual cortex and PPC (Figure 6 depicts the electrodes with significant delta/theta-HG PAC [circled in red], ERP responses [circled in black], or both [circled in yellow] for each subject). Thirty-four electrodes across the group of eight patients had both ERP responses at trial onset and significant delta/theta-HG PAC (Figure 6, electrodes circled in yellow). Thus, 44.16% (34/77) of electrodes with an ERP response also had significant increases in delta/theta-HG PAC, while 27.64% (34/123) of electrodes with significant increases in delta/theta-HG PAC also had an ERP response. Electrodes with a visually evoked ERP time-locked to trial onset were more likely to also have significant PAC throughout the attentional allocation period, while electrodes with significant PAC throughout the attentional allocation period were not as likely to be accompanied by ERP responses at trial onset. The small percentage of parietal and extrastriate electrodes with both an ERP and significant delta/theta-HG PAC throughout the attentional allocation period provides further evidence that the PAC results were not solely driven by trial onset ERPs.

Next, we averaged the signals from all electrodes that exhibited individual ERPs at trial onset (*n* = 77) and target onset (*n* = 29). There were no significant differences between the attended and unattended conditions in the P1, N1, or P3b components of the ERP time-locked to trial onset across the group (all *p*>0.10, corrected; Figure 5b, top left). The P1 and N1 components of the ERP time-locked to target onset were not significantly different between the attended and unattended conditions across the group, although attended trials evoked a marginally significantly higher P1 than unattended trials (all *p*>0.05, corrected; Figure 5b, top right). However, the P3b amplitude was significantly higher in the attended condition than the unattended condition 280–580 ms following target appearance (all *p*<0.05, corrected; Figure 5b, top right, blue shaded area), which is consistent with previous studies [31],[34]. The individual electrode example reflects the group results (Figure 5a, top right). These results suggest that the ERPs generated at trial onset were due to the onset of the display, and did not differentiate between stimulus conditions, while the target responses were modulated by spatial attention.

We next examined the ERPs from the same conditions (attended/cued vs. unattended/uncued, time-locked to trial and target onsets) averaged across all of the electrodes with significant delta/theta-HG PAC (*n* = 123; Figure 5b, bottom). These ERPs were diminished (in the case of the trial onset ERP) or nearly absent (in the case of the target onset ERP). The lack of an ERP at target onset in these electrodes suggests that the significant increases in delta/theta-HG PAC are not always accompanied by the presence of a visually evoked ERP, indicating that the target onset ERP does not contribute to the observed PAC increases in the pre-target interval.

**Figure 7:**
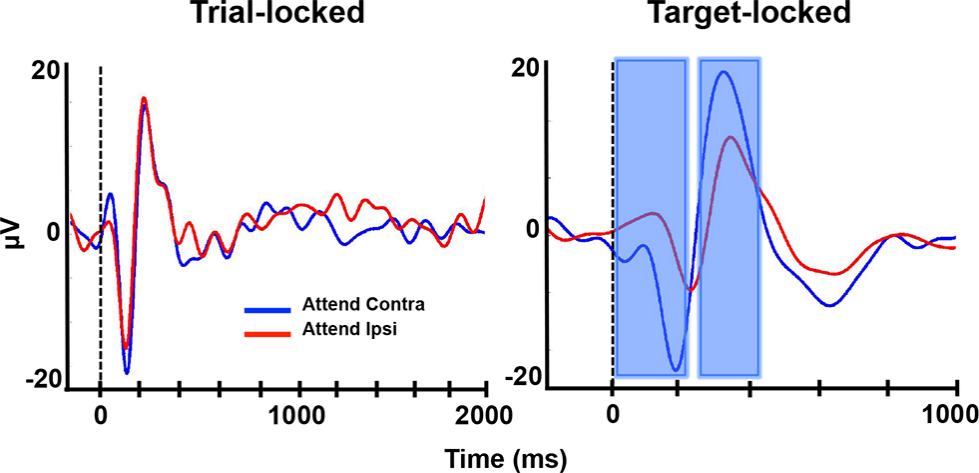
ERPs: Attention to contralateral vs. ipsilateral visual field. ERPs averaged across all trials where the attended target appeared in either the visual field contralateral (blue traces) or ipsilateral (red traces) to the implanted hemisphere and time-locked to trial onset (left) or target onset (right). ERPs are averaged across all electrodes that showed an individual ERP (*n* = 77). All other conventions are the same as in Figure 5.

We also examined separate ERP responses for trials when attention was directed to either the contralateral or ipsilateral visual field averaged across all electrodes that exhibited individual trial-locked (*n* = 77) or stimulus-locked (*n* = 29) ERPs. Again, there were no significant differences between the contralateral and ipsilateral attentional conditions for any of the ERP components time-locked to trial onset (all *p*>0.05, corrected; Figure 7, left), while N1 and P3b amplitudes were enhanced when the attended target appeared in the contralateral visual field in comparison to when it appeared in the ipsilateral visual field (all *p*<0.05, corrected; Figure 7, right). These results are consistent with previous studies showing enhanced N1 and P3 amplitudes following allocation of spatial attention to the contralateral visual field [33],[34] and provide electrophysiological evidence that subjects were allocating spatial attention during the task.

Taken together, these results suggest that the relationship between the visually evoked ERPs that are generated over PPC and visual cortex and the observed increases in PAC over these same areas is complex. Many electrodes had significant PAC without an ERP and vice versa, providing evidence that PAC and ERPs result from distinct cortical mechanisms, with ERPs reflecting post-synaptic potentials and HG power reflecting neuronal firing rates and the synaptic currents induced by this firing [12],[37].

## Discussion

We used ECoG recordings in humans to provide insight into the neural mechanisms supporting visual attention. We found increases in broadband HG power (70–250 Hz) time-locked to trial onset that remained elevated throughout the attentional allocation period over frontal, parietal, and visual areas. Attention modulated broadband HG power in two distinct patterns. First, ∼20% of these electrodes exhibited visual field-dependent attentional increases in HG (stronger in the contralateral than ipsilateral visual field), which is consistent with visual field-dependent attentional increases in single-unit activity observed in primates [2],[3]. Second, the remaining electrodes exhibited task-dependent HG increases that were independent of attended visual field, which is consistent with the broadly tuned, bilateral receptive field properties of the neurons over which we recorded [28]. The phase of the ongoing delta/theta (2–5 Hz) oscillation modulated these HG power changes during attentional allocation and, importantly, the strength of the delta/theta phase-HG amplitude coupling predicted RTs to detected targets on a trial-by-trial basis.

The current study provides evidence that coupling between HG amplitude and the phase of the delta/theta signal serves as a mechanism to facilitate processing within frontal, parietal, and visual areas during allocation of visuospatial attention. Based upon evidence from animal neurophysiology, we hypothesize that the PAC identified in the current study may serve to coordinate spiking activity in local regions of the network [38]. Phase coherence, in comparison, has been hypothesized as a potential mechanism through which distant brain areas are engaged across task-relevant networks. This synchronization of neuronal oscillations between areas may provide a mechanism for attentional selection of relevant sensory stimuli [14]. Numerous studies in humans and macaque monkeys have reported coherence increases across a wide range of band-limited frequencies, including delta, theta, alpha, beta, and low gamma, within and between frontal, parietal, and visual cortex during visual attentional allocation [6],[7],[9],[23],[39]–[46]. Thus, we hypothesize delta/theta-HG PAC increases that we observe in local regions of the fronto-parietal attention network may be modulated by neural synchrony across the network using one or more of these frequency bands [47], perhaps through a series of nested oscillations [48].

Our results are compatible with those reported by Lakatos and colleagues [43], who investigated PAC in V1 while monkeys were trained to attend to either visual or auditory stimuli presented in a rhythmic stream. They found that neural activity entrained to the stimulus rhythm (1.55 Hz) and that the amplitude of the multi-unit responses, and LFPs were systemically related to the phase of this delta oscillation during attention to both modalities. A study using a similar rhythmic attentional paradigm found coupling between delta phase (1–3 Hz) and alpha amplitude in human auditory cortex using ECoG, but amplitudes at higher frequencies were not significantly coupled to delta phase [23]. Several recent studies have also reported increases in coupling between gamma power and alpha phase in visual areas V4 and TEO while monkeys performed a flanker task [46], and between broadband HG power and beta phase over occipital cortex while humans performed a visual search task with free viewing [30].

A number of factors may contribute to differences in the observed results among the aforementioned studies. First, it is not clear whether attention operates using a mechanism at similar frequencies across modalities. For instance, oscillatory mechanisms underlying auditory attention (i.e., [23]) might be different from those underlying visual attention. Second, it is not clear whether PAC would operate at similar frequencies over fronto-parietal and visual cortex in the monkey and the human during attentional allocation. Although gamma oscillations are associated with attentive aspects of visual processing in monkey [39],[40] and human [49]–[51] cortex, considerably less evidence exists to support functional similarities among lower frequency oscillations between species. Given the size difference between the monkey and the human brain, interactions within and between lower frequency bands may differ between species. Third, each of these studies investigated attention using different types of tasks (detection vs. flanker vs. spatial cuing) and different trial structures. Many previous studies were not designed to investigate lower-frequency rhythms (i.e., 2–5 Hz), since the cue-target time (the attentional allocation period) was not sufficiently long enough to include 2–3 cycles. We purposely designed our experiment with a long interval between cue and target, so that we could investigate effects at lower frequencies. Furthermore, some experiments have investigated synchronization and PAC using entrainment to rhythmically presented stimuli [23],[43],[45] or using a fixed interval [30], while others, including the current study, have used temporally jittered target presentations. Thus, many of the previous studies have focused on how networks can be entrained by the cadence of a task, whereas the slower rhythms that we report here appear to be endogenous and are most likely determined by the local neurophysiology. It was previously suggested that lower-frequency oscillations might be suppressed when attention is utilized to detect unpredictable target appearances, since this requires a mode of operation with continuously high excitability and vigilance [52]. The results from the current study suggest that this is not the case; lower-frequency oscillations are utilized for sensory processing and attentional control, even in the absence of any obvious task-relevant temporal structure. Finally, the recording locations are different between studies. Most of these studies have investigated neuronal oscillations over sensory cortex during attentional allocation. The current study investigates how frontal and parietal association cortices utilize PAC during attentional allocation in the human brain. In summary, it is evident based upon these studies, that no single frequency band is solely responsible for attentional processes, in accord with the notion that attention is not a unitary function [53].

ERP responses at trial or target onset cannot explain these PAC results. First, there were few electrodes that had both visually evoked ERPs time-locked to trial onset and increases in delta/theta-HG PAC during the attentional allocation period (Figure 6). Many of the electrodes that were responsive to the Starry Night Test showed independent ERP responses or PAC increases. This suggests that ERPs and PAC convey different types of informational content and are dependent upon distinct underlying cortical dynamics. These results support previous studies that have reported a separation between ERPs, broadband HG, and PAC across multiple cortical regions [30],[54],[55]. Secondly, we did not observe any systematic signal changes immediately preceding target appearance in the single trial ERPs (Figure 5a) or in the average ERPs, in any electrode. Thus, it is unlikely that ERPs at target onset were responsible for the observed PAC increases in the pre-target interval. Lastly, delta/theta-HG PAC remained significantly elevated in nearly all electrodes after eliminating the initial 500 ms (when the ERP was generated) from the longest (>1500 ms) trials (Figure S2). Together, these results suggest that the observed increases in delta/theta-HG PAC are due to the allocation of visuospatial attention, rather than an artifact of analysis procedures.

A recent study in macaque monkeys reported that microsaccades occur at a frequency of ∼3.3 Hz and are followed by characteristic increases and decreases in gamma band synchronization [56]. We cannot completely rule out the possibility that the increases in delta/theta-HG PAC were a result of microsaccadic activity, since our eye-tracker did not have sufficient sensitivity to measure microsaccades and hospital protocols did not allow patient head stabilization in the epilepsy ICU environment. We think this case is unlikely, since microsaccades induce bilateral cortical effects [57], while many of the electrodes exhibited strongly lateralized increases in delta/theta-HG PAC. Furthermore, microsaccades were shown to modulate spike-field coherence at lower gamma frequencies, 40–60 Hz [56], while our PAC results were strongest above 70 Hz. Microsaccades are present during most tasks that require sustained fixation [58] and several studies have indicated a close relationship between microsaccades and shifts in visuospatial attention [59],[60]. Several recent studies have provided behavioral evidence that attentional switching occurs between visual field locations or objects at ∼4 Hz rhythm [61],[62]. We propose that delta/theta-HG PAC may reflect an underlying neural mechanism that regulates the timing of such rhythmic sampling across the visual field or across objects.

The current study demonstrates a functional relationship between PAC and behavioral performance in the SNT: the strength of delta/theta phase-HG amplitude coupling predicted RTs on a trial-by-trial basis, wherein high PAC predicted faster RTs and low PAC predicted slower RTs during attentional allocation. Similar coupling between theta phase and gamma amplitude has been tied to learning in the rodent hippocampus [63]: the strength of theta-gamma PAC increases as a rodent's performance increases, both plateauing together, over time. These studies are important, since they provide evidence that PAC serves a functional role in the brain. The current study demonstrates that delta/theta phase-HG amplitude coupling also serves a functional role by indexing the overall level of engagement within human frontal and parietal cortex during the allocation of visuospatial attention.

## Materials and Methods

### Subjects

Eight subjects (S1–S8), undergoing pre-surgical epilepsy evaluation, provided written and oral informed consent to participate in the study. The institutional review board of each institution approved the research that was conducted at each respective location. Anti-epileptic medications were discontinued for 2–3 days beforehand, and patients were seizure free for at least five hours before testing. Subjects had normal or corrected-to-normal vision.

Subjects were implanted with 74–128 electrodes (1 cm spacing; grids and strips), covering extensive portions of lateral and medial frontal, parietal, occipital, and temporal cortex in the RH and LH (see Figure 1a for overlap of electrodes from all subjects and Table 1 for specific coverage information for each subject).

### Visual Display, Stimuli, and Experimental Design

Visual displays were generated on a Dell Precision M4600 laptop (Dell, Inc.) using EPrime software (Psychology Software Tools, Inc.). Subjects were seated 50–60 cm from the computer screen.

#### Starry Night Test

In an adapted version of this task [24], subjects attended either to the LVF or RVF and fixated on a cross in the middle of the screen during each trial. On each trial (42 total per block), subjects covertly attended to the instructed visual field (center arrow cue) and pressed a button when a blue square target appeared (6.5 mm^2^ in size with an average luminance of 34.28 cd/m^2^) (Figure 1b). Target onset was jittered from 1000–2000 ms after trial onset. The target remained on the screen until the subject responded, followed by a 500 ms intertrial interval (ITI). Subjects withheld responses for blue squares in the unattended field. An unattended stimulus remained on the screen for 2000 ms, followed by a 500 ms ITI. Six out of the eight subjects responded using the hand ipsilateral to the grid.

The screen was divided into a 6×7 grid containing 42 virtual cells (grid not visible). Targets appeared ∼62% on the cued/attended side and ∼38% on the uncued/unattended side. Targets appeared on a dynamic background of red circle distracters (each 4 mm in diameter with an average luminance of 64.98 cd/m^2^), which could turn on (visible) or off (invisible). One red distractor located anywhere in the screen turned on or off with random intervals of 50–250 ms, hence the name “Starry Night”. A 2 (attention: attended vs. unattended)×2 (visual field: contralateral to grid vs. ipsilateral to grid) design was employed, with a total of 6 blocks (156 trials/attended, 96 trials/unattended) from each subject. This task was similar to a Posner cuing paradigm, except: (1) targets could appear anywhere in a hemi-field, permitting investigation of parafoveal and peripheral attention, and (2) dynamic distracters enhanced the sensitivity of the test by increasing attentional competition.

Underneath a photodiode placed at one corner of the screen, a white square appeared once at trial onset and again at target onset and disappeared following subject response to mark events and RTs.

### Data Acquisition

Electrophysiological and the peripheral (photodiode) channels were acquired using a 128-channel Stellate Harmonie recording system (Natus Medical, Inc.; 1000 Hz digitization) at Johns Hopkins, a 128-channel Nihon Kohden recording system (Nihon Kohden Corporation; 1000 Hz digitization) at Children's Hospital, and 128-channel Tucker Davis Technologies recording system at Stanford (3052 Hz digitization). Data were recorded using a subdural electrode reference and a scalp ground.

### Electrode Localization

Postoperative computed tomography (CT) scans were aligned to the preoperative MRI anatomical brain volume [64]. Electrodes were visualized on the 3D cortical surface using MATLAB (MathWorks, Inc.; Figures 2–5). Brains and electrodes were transformed into MNI space across subjects (Figure 1a, Figure S1).

### Data Analysis

A neurologist manually inspected all ECoG channels to identify those with interictal or ictal epileptiform activity and artifacts. Channels and epochs contaminated by epileptiform activity or abnormal signal (e.g., poor contact, excess drift, high frequency noise) and those located over tissue that was later resected were removed from analysis.

Data processing used custom functions written in MATLAB and the EEGLAB toolbox [65]. Raw, continuous data were filtered with a 60 Hz notch filter and re-referenced to a common average reference (mean of remaining channels). Single channels of this ECoG data are referred to as “raw signal.”

Trials were classified based upon attention condition (attended target in cued visual field; or unattended target appearing in the uncued visual field). Contralateral and ipsilateral attention conditions refer to the cued visual field with respect to the implanted ECoG electrodes. All trials were designated by response type (hits, misses, correct rejections, and false alarms). Correctly attended trials were designated hits and correctly unattended trials were designated rejections.

#### Spectral decomposition

The ECoG was filtered in each frequency range using a two-way least-square finite impulse response filter [65] to prevent phase distortion. The Hilbert Transform was applied to these filtered signals to create a complex-valued analytic time series. Squaring the modulus (element-wise) of the complex-valued analytic time series created the instantaneous power time series. Taking the angle (element-wise) of the complex-valued analytic time series created the instantaneous phase time series.

#### Event-related spectral perturbations

ERSPs were calculated for each electrode (Figure 2). Forty-four logarithmically equally spaced frequency bands were created from 1 Hz to 250 Hz. The instantaneous power time series was calculated for each frequency band and ERSPs were created for each condition by averaging the power time series across all trials from that condition (0 ms to 2000 ms post-trial-onset). A bootstrapped distribution of baseline values was created by randomly choosing *N* ( = number trials/condition) baseline values and averaging across these values (for 1000 iterations). The ERSP was normalized (per frequency) by subtracting the mean of the baseline distribution and dividing by the standard deviation (SD) of the baseline distribution (*z*-score). The red and blue portions of each graph are at least ± two z-scores (*p*<0.05), respectively, from the mean of the baseline. Electrodes were counted as having significant HG increases if HG power (70–250 Hz) was significantly above baseline following trial onset and was sustained for at least 750 ms thereafter, as indicated by the ERSP. Electrodes were counted as having significant alpha/beta decreases (suppression) if alpha/beta power (10–30 Hz) was significantly below baseline following trial onset and was sustained for at least 500 ms thereafter, as indicated by the ERSP. Differences between the contralateral and ipsilateral attention conditions were determined by subtracting the ipsilateral ERSP from the contralateral ERSP, first assuring equal variances and trial numbers between conditions.

#### Single-trial HG traces

The instantaneous HG power (70–250 Hz) time series was calculated for each trial and normalized to its own baseline (−250 to −50 ms pre-trial onset) (Figure 2). Each trace is locked to trial onset and ends 2000 ms later. Black tick marks represent target onset for each trial. Trials were stacked from shortest to longest target onset.

#### PAC analysis

Electrodes with significantly greater HG (70–250 Hz) power following trial onset compared to baseline were used in the PAC analyses (see below). Post-trial onset power was defined as averaged power across a 500 ms window (50 to 550 ms) and baseline power was defined as averaged power across a 500 ms window before trial onset (−550 to −50 ms). Significance was assessed using a single-tailed repeated-measures *t*-test, assuming unequal variances, comparing baseline power with post-trial-onset power across single trials. To control for multiple comparisons, the *p*-values from each *t*-test were corrected using the False Discovery Rate (FDR [66]) and considered significant at *q*<0.01.

PAC was estimated using the phase-locking value [67] and the procedure outlined by Voytek and colleagues [68]. For a pair of frequencies, the raw signal from each electrode was filtered twice: once at a lower frequency (f_1_) of interest and once at a higher frequency (f_2_) of interest. The instantaneous phase was calculated for the f_1_ time series. The analytical amplitude of the f_2_ time series was then refiltered at the lower frequency (f_1_) and the instantaneous phase was also calculated for this time series. The phase time series were then separated into different trial types (attend contralateral or attend ipsilateral, 0–1000 ms post-trial onset) and a single PLV was calculated across all trials for each electrode and condition.

Comodulograms were created for individual electrodes that passed the above selection criterion. PLVs were calculated for a range of frequencies for the phase data (2–20 Hz, in 2 Hz increments) and a range of frequencies for the amplitude data (5–250 Hz, in 2 Hz increments) and plotted (frequencies for phase, x-axis; frequencies for amplitude, y-axis; Figure 3a,b). The comodulogram contours correspond to PLVs associated with *p*-values (*p* = 0.50, 0.10, 0.05, 0.01, 0.005). For each comodulogram, we calculated the five PLVs that corresponded to these five *p*-values. A surrogate distribution of 200 PLVs was created for each frequency pair. For each iteration, the phase time series was shifted forward or backward by a randomly chosen integer lag and the PLV was recalculated. Next, the 200 PLVs for each frequency pair were fit with a gamma distribution and the parameter estimates (shape, scale) were determined for that pair. PLVs corresponding to probabilities of 0.995, 0.990, 0.950, 0.900, and 0.500 were calculated for each frequency pair using the inverse of the gamma cumulative distribution function. We chose to use the values from the frequency pair that yielded the most conservative gamma distribution (i.e., the largest PLVs) as estimates for each of the *p*-values. Separate comodulograms were created for attend contralateral and attend ipsilateral conditions.

We used these comodulograms to focus on two specific frequency ranges, one for the phase data (2–5 Hz) and one for the amplitude data (100–150 Hz), to assess PAC significance in each electrode. A single PLV was calculated for each electrode and trial type and compared to a surrogate distribution of 1000 PLVs. The phase time series was shifted forward or backward by a randomly chosen integer lag and the PLV was recalculated for each iteration. A *p*-value was determined from this surrogate distribution and corrected using an FDR procedure [66] (significant at *q*<0.01). A Mann-Whitney *U*-test was performed across subjects to determine whether the number of electrodes with significant delta/theta-HG PAC differed between hemispheres.

To determine whether PAC was stronger in contralateral than ipsilateral attention conditions, the PLV was calculated for each attend contralateral trial and each attend ipsilateral trial (averaged across 0–1000 ms post-trial onset) in each of the electrodes that passed the above selection criterion. Single-tailed two-sample *t*-tests, assuming unequal variances, were used to assess statistical significance between attend contralateral and attend ipsilateral trials in each electrode. *p*-Values were FDR-corrected [66] across electrodes (significant at *q*<0.01). To address whether PAC differences were due to power differences between conditions [29], we compared the magnitudes of delta/theta (2–5 Hz) power averaged across the same time period (0–1000 ms post-trial onset) between attend contralateral and attend ipsilateral trials. Delta/theta power was calculated for each trial in each attention condition in each of the electrodes with significant PAC differences between attend contralateral and attend ipsilateral conditions (*n* = 55). In each electrode, two-sample *t*-tests, assuming unequal variances, assessed statistical significance of power values across trials between the two conditions and *p*-values were FDR-corrected [66] (significant at *q*<0.05).

To address the contributions of trial onset ERPs, we repeated all of the above analyses (comodulograms, significance testing, PAC and power differences between conditions) using only the longer trials (i.e., those where the target appeared 1500–2000 ms after trial onset). Only the last 1000 ms prior to the target onset were analyzed (−1000 to 0 ms prior to target onset). As a result, the first 500 ms of each trial, where the ERP occurred, was excluded.

#### PAC–behavior correlations

A single PLV between delta/theta phase (2–5 Hz) and HG amplitude (100–150 Hz) was calculated (averaged across −1000 to 0 ms pre-target onset, to exclude any ERP effects) for attend contralateral and attend ipsilateral trials, in electrodes with significant delta/theta-HG PAC (*n* = 123). The Pearson product-moment correlation coefficient was calculated between PLVs and RTs across trials and linear regression lines were fit for each condition and each electrode (e.g., Figure 4). Trials were excluded from the correlation analysis if RTs were greater than three SDs above the mean. Similar correlations were calculated between RTs and mean amplitude values of the delta/theta (2–5 Hz) and HG (100–150 Hz) signal averaged across the same time period used above. All *p*-values were FDR-corrected [66] across electrodes (significant at *q*<0.05).

#### Trough-locked spectrograms

Trough-locked ERPs of the filtered delta/theta signal (2–5 Hz; Figure 3c, bottom) ±1000 ms and the corresponding time points of the normalized instantaneous power time series (1–250 Hz; trough-locked spectrogram; Figure 3c, top) were created following the procedure outlined by Canolty and colleagues [19]. Only time points from attention trials (both contralateral and ipsilateral trials; 0–2000 ms post-trial-onset) were included. Only electrodes that passed the PAC electrode selection criterion were included in this analysis.

#### ERP analysis

The raw time series for each electrode were filtered between 0.1 and 8 Hz, each trial was normalized to its own baseline (by subtracting the average of the 200 ms preceding each trial from each time point), and trials were averaged for each condition. We examined these data time-locked to the trial onset (−200 to 1000 ms) and to the target onset for two different periods: pre-target onset (−1000 to 200 ms) and post-target onset (−200 to 1000 ms). We then identified the electrodes in each subject with clear visual ERP responses that were time-locked to trial or target onset, as defined by the P1-N1-P3b complex [31],[32],[34] (Figure 5). We tested for significant differences between conditions (attended/cued vs. unattended/uncued; attend contralateral vs. attend ipsilateral) using a point-by-point two-sample *t*-test on a time window of 50–1000 ms post-trial onset and post-target onset. Multiple comparisons were corrected using the FDR procedure [66]. Blue, shaded areas throughout the figures indicated the ERP time points with significant differences across conditions. These analyses were repeated by averaging the signals across all electrodes with significant delta/theta-HG PAC (Figure 5b). Single-trial ERP traces (e.g., Figure 5a) were also created for individual electrodes, which were similar to the single-trial HG traces (described above), but each trace is the raw time series filtered between 0.1 and 8 Hz and baseline corrected (by subtracting the average of the 200 ms preceding each trial from each time point) for each trial.

We examined the ERP responses preceding target onset to determine if any preparatory activity existed leading up to the target appearance. We did not observe any systematic signal changes immediately preceding target appearance, in the single trial ERPs (e.g., Figure 5a) or in the average ERPs, in any electrode in any subject. It was therefore unlikely that subjects were anticipating the target appearance, and it was also unlikely that the target ERP was responsible for the observed PAC increases in the pre-target interval. We therefore decided to include the time points immediately preceding target onset in our analyses (e.g., for those analyses that use the time window −1000 to 0 ms pre-target onset).

#### Eye movement controls

We instructed subjects to keep their eyes on the fixation cross and we visually monitored all subjects in the epilepsy intensive care unit to confirm fixation during SNT performance. None of the subjects made systematic saccadic eye movements during task performance. To further assess potential eye movement confounds, we examined PAC in orbito-frontal cortex (OFC) and temporal pole electrodes (*n* = 48; S1, S3–S6, S8). Because of their proximity to the eye orbits, electrodes over these regions are more susceptible to eye movement artifacts [69]. If present, artifacts should be found in these electrodes during SNT performance. We did not find significant delta/theta-HG PAC during attentional allocation in any of the OFC or temporal pole electrodes (all *p*>0.10, corrected), despite finding significant PAC effects in frontal and parietal electrodes in these same patients.

In addition, we monitored the eye movements of one subject (S8) during the recording session while the subject preformed the SNT. We recorded eye position data on the stimulus presentation computer using a RED-m eye tracking system (SensoMotoric Instruments GmbH [SMI]), which measured both eye positions at a rate of 120 Hz, had a spatial resolution of ∼0.10°, and an accuracy of ∼0.50°. We used SMI BeGaze software and custom MATLAB functions to analyze the eye movement data. To study the subject's fixation accuracy and precision, we calculated frequency histograms of the vertical and horizontal eye position data separately for periods of attention to each hemifield and then used two sample *t*-tests to determine whether eye movements systematically deviated in any direction during each condition. We additionally defined several regions of interest, 1°×1° or 1.5°×1.5° in size, around the central fixation cross and calculated the number of times the gaze left this region. The maximum difference in mean horizontal and vertical eye positions among the experimental conditions was 0.17° and 0.10°, respectively. There were no systematic horizontal or vertical eye deviations between any of the experimental conditions (all *p*>0.30). Fixation was well maintained and very rarely left the area surrounding the central fixation cross. 93.30% and 95.62% of the horizontal and vertical position samples during the experiment were located within the 1°×1° and 1.5°×1.5° regions of interest, respectively. This confirms that S8 maintained fixation throughout the experiment and did not shift gaze location along with attention.

We excluded from any further data analyses all trials during which S8 made a saccadic eye movement of 0.50° or larger. We observed significant increases in delta/theta phase-HG amplitude coupling in numerous electrodes, even after the trials containing the saccades were removed (see Figure 6 for details on these electrodes in S8). This demonstrates that saccadic eye movements (at least those larger than 0.5°) were not responsible for the observed PAC effects.

#### Behavioral measures

The offset of each target occurred at the same time as a subject's manual response (in the case of attended trials) and at the end of 2000 ms (in the case of unattended trials). RTs for the attended trials were calculated based upon the difference between the offset and onset times of each target, measured by the photodiode. RTs and accuracy (% hits, % correct rejections) were averaged across trials and conditions and *d′* was calculated for each subject. One-way ANOVAs and repeated-measures *t*-tests were used to analyze the behavioral results.

## Supporting Information

Figure S1 **PAC group results.** Average amount of PAC (as measured by the PLV) during attentional allocation (0–1000 ms post-trial onset) for each electrode across the group of subjects (*n* = 8). Group results were plotted separately on the right and left hemispheres (right, left, respectively) of the MNI standardized brain.
(TIF)

Figure S2 **Delta/theta phase-HG amplitude coupling in long trials.** PAC was re-calculated using only trials with a long attentional delay period (>1500 ms). The resulting comodulograms are presented for the same electrode and subject examples as in Figure 3. All other conventions are the same as in Figure 3a,b.
(TIF)

## Abbreviations

ECoG: electrocorticography
EEG: electroencephalography
ERP: event-related potential
ERSP: event-related spectral perturbation
FDR: False Discovery Rate
fMRI: functional magnetic resonance imaging
IPS: intraparietal sulcus
HG: high gamma
LFP: local field potential
LVF: left visual field
OFC: orbito-frontal cortex
PAC: phase-amplitude coupling
PLV: phase-locking value
PPC: posterior parietal cortex
RT: reaction time
RVF: right visual field
SD: standard deviation
SNT: Starry Night Test
