# Supplementary figures and images for: Dynamic Changes in Phase-Amplitude Coupling Facilitate Spatial Attention Control in Fronto-Parietal Cortex
Source: PLoS Biol. 2014 Aug 26;12(8):e1001936. doi: 10.1371/journal.pbio.1001936 (PMC4144794; doi:10.1371/journal.pbio.1001936)

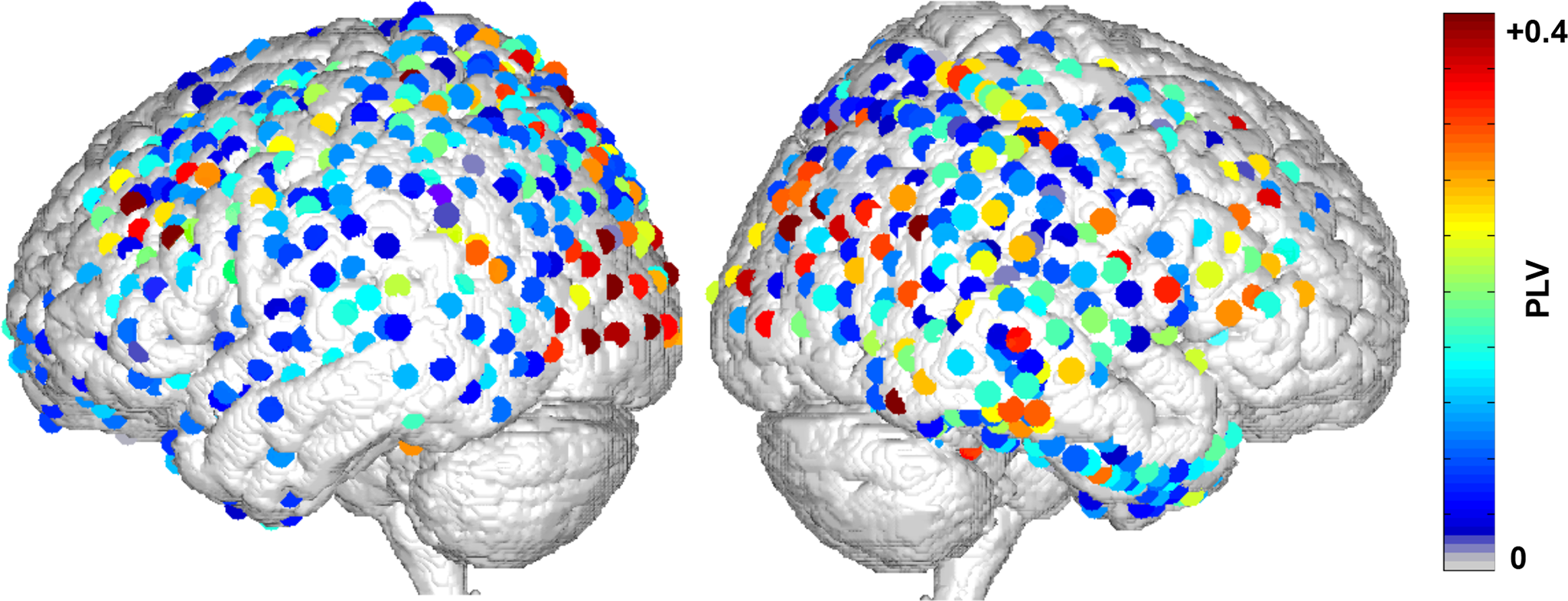

Supplement: Figure S1 — PAC group results. Average amount of PAC (as measured by the PLV) during attentional allocation (0–1000 ms post-trial onset) for each electrode across the group of subjects (n = 8). Group results were plotted separately on the right and left hemispheres (right, left, respectively) of the MNI standardized brain. (TIF) [file pbio.1001936.s001.tif]

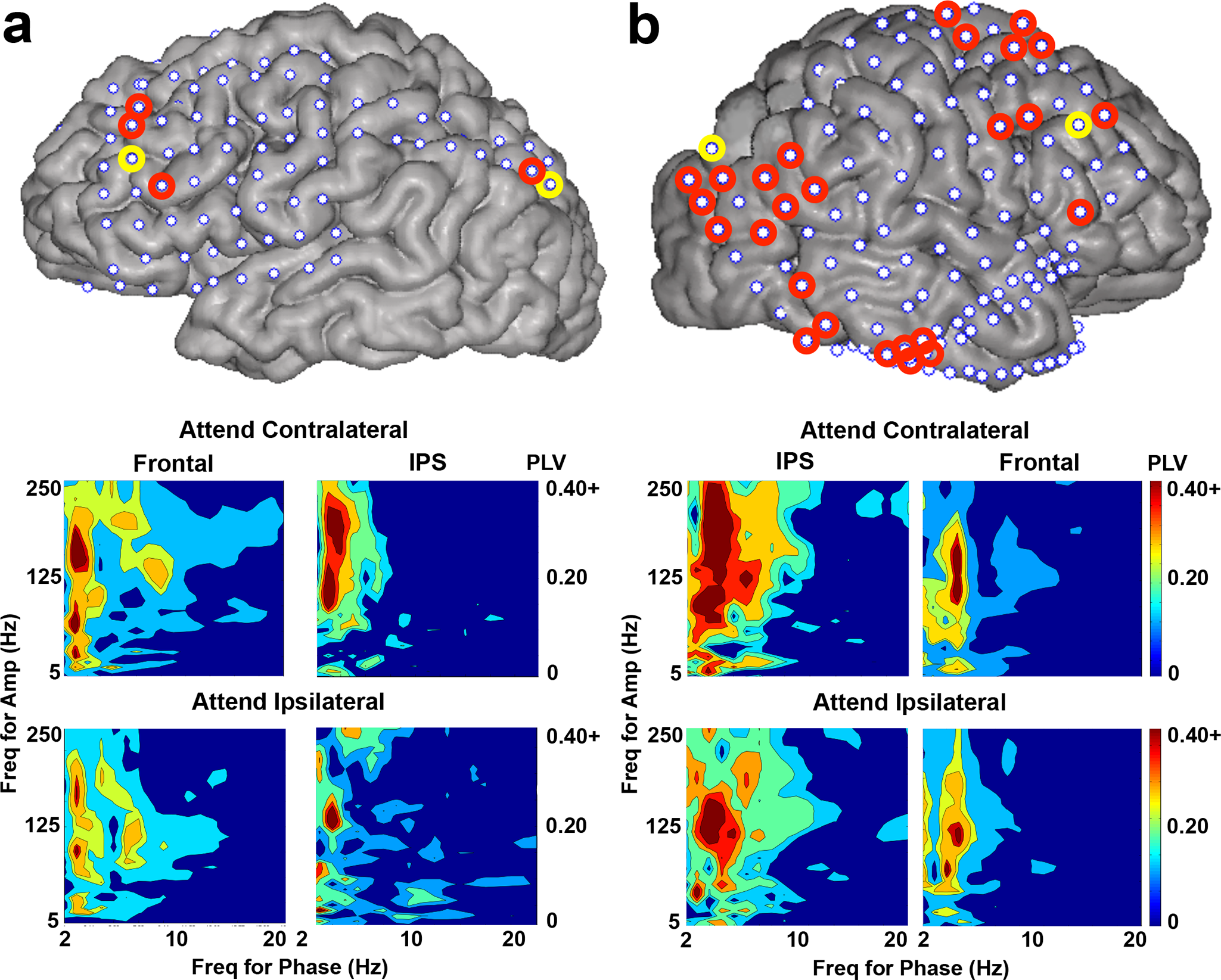

Supplement: Figure S2 — Delta/theta phase-HG amplitude coupling in long trials. PAC was re-calculated using only trials with a long attentional delay period (>1500 ms). The resulting comodulograms are presented for the same electrode and subject examples as in Figure 3. All other conventions are the same as in Figure 3a,b. (TIF) [file pbio.1001936.s002.tif]
